# Antitumor Activity of Novel Transcriptional Inhibitors Ecubectedin and PM54 in Soft Tissue Sarcoma Patient-Derived Xenografts

**DOI:** 10.1158/2767-9764.CRC-26-0377

**Published:** 2026-09-02

**Authors:** Daniël Gorgels, Chao-Chi Wang, Lore De Cock, Luna De Sutter, Karo Wyns, Kimberly Verbeeck, Ulla Vanleeuw, Daphne Hompes, Friedl Sinnaeve, Hazem Wafa, Maria Jose Guillén, Pablo Avilés, Raf Sciot, Agnieszka Wozniak, Patrick Schöffski

**Affiliations:** 1 Laboratory of Experimental Oncology, Department of Oncology, Leuven Cancer Institute, KU Leuven, Leuven, Belgium.; 2 Department of General Medical Oncology, University Hospitals Leuven, Leuven Cancer Institute, Leuven, Belgium.; 3 Department of Surgical Oncology, University Hospitals Leuven, Leuven Cancer Institute, Leuven, Belgium.; 4 Department of Orthopedic Surgery, University Hospitals Leuven, Leuven Cancer Institute, Leuven, Belgium.; 5 PharmaMar S.A., Madrid, Spain.; 6 Department of Pathology, University Hospitals Leuven, Leuven, Belgium.

## Abstract

Trabectedin, prototype of the ecteinascidin class of drugs, is a known second- or later-line therapeutic option for advanced soft tissue sarcoma (STS). We evaluated the antitumor activity of two novel synthetic trabectedin derivatives, ecubectedin and PM54, in selected patient-derived xenograft (PDX) STS models. In total, 364 Naval Medical Research Institute^*nu/nu*^ mice were transplanted bilaterally with two leiomyosarcoma (LMS), two dedifferentiated liposarcoma (DDLPS), one synovial sarcoma (SynSa), and one *CIC*-rearranged sarcoma (CRS) PDX models. Mice were randomized to six groups and treated via tail vein injection with (i) vehicle, (ii) doxorubicin, (iii) trabectedin, (iv) lurbinectedin, (v) ecubectedin, or (vi) PM54. Treatment was given on days 1, 8, and 15, and mice were sacrificed on day 16. The SynSa experiment included extra mice to investigate posttreatment xenograft evolution. Antitumor activity was assessed by tumor volume measurement, histopathologic, and immunohistochemical analyses. In all LMS and DDLPS models, ecubectedin and PM54 led to tumor growth delay compared with trabectedin. Histopathologic evaluation of treated tumors showed moderately increased antitumor activity of novel ecteinascidins compared with trabectedin. The CRS model showed tumor shrinkage in response to ecubectedin (63% regression from baseline) and PM54 (24% regression from baseline), while the SynSa model showed tumor volume stabilization. Both translocation-related models showed significant antitumor effect on histopathologic evaluation in response to the novel drugs compared with trabectedin. Ecubectedin and PM54 have modest antitumor activity in STS PDX models, with the strongest effects seen in the translocation-related sarcoma models, showing the potential of this class of drugs in the treatment of STS.

**Significance:**

Advanced STS remains without truly effective therapeutic options. Translocation-related sarcomas demonstrate higher vulnerability to next-generation ecteinascidin treatment compared with more complex sarcoma subtypes. This vulnerability builds upon known literature and could be exploited in future clinical trials using the next-generation ecteinascidins.

## Introduction

Soft tissue sarcomas (STS) are a very heterogeneous group of malignant solid tumors arising from mesenchymal progenitor cells (1). STSs are rare diseases, collectively accounting for <1% of all adult malignant solid tumors, with an incidence rate of 3 to 4/100,000 persons per year globally (1). The heterogeneity of STSs is emphasized by the histologic and molecular characterization of >80 subtypes in the most recent version of the World Health Organization classification (2). From the genetic perspective, the minority of STSs (∼20%) are driven by specific genetic alterations, usually with subtype-specific translocations (translocation-related sarcoma), or activating mutations. These sarcomas usually present with a simple karyotype. The majority of STS subtypes are characterized by a complex karyotype, with multiple numerical and structural chromosomal alterations (3, 4). The 5-year overall survival for patients with STS across all subtypes and disease stages is around only 50% (5). For patients with advanced or metastatic disease, the 5-year overall survival rate is closer to 10%, with a median survival of 12 months in historic series (1, 6). For more than 40 years, doxorubicin monotherapy still remains the standard of care for advanced or metastatic STS, despite showing response rates of less than 15%, providing poor disease control, and being associated with poor survival outcome and dose-limiting cardiotoxicity (6). Thus, current treatment results remain highly unsatisfactory and illustrate a dire need for improved therapeutic options.

Trabectedin (PharmaMar) is approved and commonly used in patients with advanced STS after failure of doxorubicin. Trabectedin belongs to the class of ecteinascidins, originates from the marine tunicate *Ecteinascidia turbinata*, and is now produced synthetically. Trabectedin is an alkylating agent, binding to guanine in the minor-groove of DNA (7). The drug has a unique mechanism of action, manipulating the transcription-coupled nucleotide excision repair machinery of cells to generate single-strand DNA breaks, which convert into DNA double-strand breaks and lead to cell death (8–11). In the United States, trabectedin is approved for the second-line treatment of liposarcoma (LPS) and leiomyosarcoma (LMS; ref. 12). In Europe, trabectedin is approved as second-line treatment of all STSs after failure of anthracycline therapy (13). Retrospective analyses found that trabectedin has preferential activity in patients with translocation-related sarcomas such as synovial sarcoma (SynSa) or myxoid liposarcoma (14, 15).

PharmaMar has developed a synthetic derivative of trabectedin called lurbinectedin (16), which has already shown clinical activity in platinum-resistant ovarian cancer and has been approved as a second-line therapy for patients with small cell lung cancer (17–19). In STS, lurbinectedin has shown limited response as monotherapy (20). However, a combination of lurbinectedin with doxorubicin is currently being investigated in a phase IIb/III trial in patients with metastatic LMS as a first-line therapy and compared with the standard-of-care doxorubicin monotherapy (NCT06088290).

More recently, two synthetic ecteinascidins, namely ecubectedin (PM14) and PM54, have been developed by PharmaMar (21). These synthetic derivatives are designed to maintain the antitumor activity of the parent compound trabectedin, while potentially exhibiting increased potency and improved pharmacokinetics. These novel synthetic ecteinascidins have shown potent antitumor effects in melanoma *in vitro* studies and cell-derived xenograft models, as well as in refractory malignant pleural mesothelioma (21, 22).

The previously described activity of trabectedin and lurbinectedin in mesenchymal malignancies led us to speculate on the potential antitumor activity of these novel compounds in STS. This prompted the current preclinical study, in which we explored the anticancer effect of ecubectedin and PM54 in selected patient-derived xenograft (PDX) models of STS. Trabectedin was included as a benchmark because it is the prototype of the ecteinascidin class of drugs. Lurbinectedin, an approved second-generation ecteinascidin, was also included as a comparator for the new compounds.

## Materials and Methods

### Xenograft models

Six models were selected from the XenoSarc platform of PDX models, established in the Laboratory of Experimental Oncology, KU Leuven (23) for *in vivo* evaluation of ecubectedin and PM54. All models were chosen based on the STS subtype. We selected four models based on historic data of trabectedin in these subtypes: LMS (UZLX-STS22_2 and -STS111) and dedifferentiated liposarcoma (DDLPS; -STS204 and -STS112). In addition, we selected a model of SynSa (-STS336, harboring the *SS18::SSX2* fusion) and a xenograft of a *CIC::DUX4* sarcoma (CRS; -STS134), as they are translocation-related diseases in which ecteinascidins may have preferential activity and due to the high unmet medical need in these entities. Tumor material for establishment of the xenograft models was collected during surgery from donor patients treated at the University Hospitals Leuven (UZ Leuven) after written informed consent was obtained. The models are well-characterized and show stable histologic and molecular characteristics during passaging, resembling the original donor material (23). Sample collection, xenografting and model characterization, as well as the use of the PDX models for this project were approved by the Medical Ethics Committee of UZ Leuven (projects S53483 and S66560, respectively) and were performed following the ethical guidelines set forth in the Declaration of Helsinki. The *in vivo* experiments with the PDX models were approved by the KU Leuven Ethics Committee for Animal Research (P196-2020) and were performed according to local guidelines and Belgian/European Union regulations.

### Experimental design

A total of 364 adult female partially immunodeficient, athymic Naval Medical Research Institute^*nu/nu*^ mice (Janvier Laboratories) were transplanted subcutaneously and bilaterally on the flanks under general anesthesia using an intraperitoneally injected mixture of ketamine (Dechra, 100 mg/mL) and xylazine (VMD Livestock Pharma, 20 mg/mL). Bilaterally transplanted tumors from one mouse were considered nondependent events, as proven previously (23). Once the average tumor volume reached 150 mm^3^, mice were randomized into six treatment groups with 6 to 8 mice per treatment arm (the achieved number of tumors included are shown in Supplementary Table S1): (i) vehicle (5% dextrose) 5 mL/kg; (ii) doxorubicin (D1515, Sigma-Aldrich) 5 mg/kg; (iii) trabectedin 0.15 mg/kg; (iv) lurbinectedin 0.18 mg/kg; (v) ecubectedin 1.2 mg/kg; and (vi) PM54 1.2 mg/kg. The vehicle and all ecteinascidins were provided by PharmaMar as lyophilized powder. All treatments were administered once weekly via the tail vein on days 1, 8, and 15 of the experiment. All four ecteinascidin treatments were administered at maximum tolerated dose, as determined by PharmaMar. The sample size was based on historic data, as described previously (24). The tumor volume was measured three-dimensionally by using a digital caliper three times per week under isoflurane (Piramal Critical Care) and calculated using a half-ellipsoid formula: tumor volume=π × xyz/6. To evaluate the wellbeing of the animals, the body weight was measured three times per week, and the general condition of the animals was monitored daily. Tumor volume and body weight data were normalized against measurements obtained on day 1 (baseline) i.e., $\frac{Measurement\;day\;x}{Measurement\;day\;1}\;\times \;100\%$. The mice were sacrificed 24 hours after the last treatment (day 16), and tumor fragments were collected in 4% buffered formaldehyde (diluted with phosphate-buffered saline from 37% (Thermo Fisher Scientific, 119690010) to construct formalin-fixed, paraffin-embedded (FFPE) blocks and also snap-frozen in liquid nitrogen for further histopathologic and molecular analyses.

In the experiment with the STS336^SynSa^ model, the number of mice was increased to 10 to 12 mice per treatment arm with the aim to investigate whether the treatment would exert a lasting effect after treatment discontinuation. This allowed to sacrifice half of the animals from each group in that experiment on day 16, whereas remaining animals were monitored for an additional 25 days.

### Histopathologic and molecular analyses

FFPE blocks were used to cut 4-μm sections for hematoxylin (modified Gill, VWR) and eosin (Merck; H&E) or immunohistochemistry (IHC) staining. To confirm model stability, IHC was used to confirm human origin by staining for human leukocyte antigen-A (Abcam, ab52922, RRID: AB_881225). The morphology of the tumors was checked by H&E staining. Subtype-specific markers were tested by IHC: α-smooth muscle actin (Agilent, clone 1A4, M0851, RRID: AB_2223500) for LMS, mouse double minute homolog 2 (MDM2; Thermo Fisher Scientific, 33-7100, RRID: AB_2533136) for DDLPS, CD99 (Abcam, SP119, ab227738, RRID: AB_3750615) for CRS and SynSa, and cytokeratin (Agilent, AE1/AE3, M3515, RRID: AB_2132885), epithelial membrane antigen (Cell Signaling Technology, D5K9I, 16564, RRID: AB_2798765), and transducin-like enhancer of split 1 [Abcam, EPR9386(2), ab183742, RRID: AB_3741399] for SynSa. The presence of the *MDM2* amplification was confirmed by fluorescent *in situ* hybridization [FISH; Leica Biosystems, (12q15)/SE12]. Furthermore, FISH was utilized to confirm the presence of the *SSX::SS18* (Abbott, Vysis SS18 Break Apart FISH Probe Kit) rearrangement in SynSa, as well as the *CIC::DUX4* (Zytovision, Z-2285-50) translocation in CRS.

Antitumor effects were evaluated by counting the average number of mitotic figures and apoptotic cells on H&E-stained slides in 10 high-power fields (HPF, 400-fold magnification; CH30 Olympus microscope, field of view diameter: 0.45 mm). To validate these results, IHC was done for the proliferation markers phosphorylated histone H3 (pHH3; Ser10, Cell Signaling Technology, 9701, RRID: AB_331535) and Ki-67 (Agilent, clone MIB-1, M7240, RRID: AB_2142367), as well as cleaved poly-(ADP-ribose) polymerase (cPARP; Abcam, E51, ab32064, RRID: AB_777102) as a marker of apoptosis. Mitotic and apoptotic activities were assessed by counting the average number of cells positive for pHH3 and cPARP, respectively, in 10 HPFs at 400-fold magnification. The average percentage of Ki-67–positive cells was used as the proliferation index, calculated as the average percentage in five digital images taken at 400-fold magnification using an Olympus UC30 digital camera (Olympus) as described previously (25). Finally, the presence of phosphorylated histone 2AX (γH2AX; Ser139, Cell Signaling Technology, 9718, RRID: AB_2118009) was evaluated as a marker for double-strand break formation.

### Drug preparation

All drugs obtained from PharmaMar were delivered as lyophilized powders, which were reconstituted in either sterile water (vehicle, trabectedin, and lurbinectedin) or sterile 0.9% NaCl (ecubectedin and PM54), and stock solutions were stored at −80°C. Prior to injection, stock solutions were diluted to working solutions using either sterile 5% dextrose (vehicle) or sterile 0.9% NaCl (trabectedin, lurbinectedin, ecubectedin, and PM54). Doxorubicin was dissolved in sterile 0.9% NaCl at 1 mg/mL and stored at −80°C as a working solution until the day of use. Immediately before injection, all drugs were brought to room temperature and briefly vortexed.

### Statistical analysis

Relative tumor volumes within treatment groups between first and last day were compared using the Wilcoxon matched-pairs test. The Mann–Whitney U test was used to compare relative tumor volumes of different treatment groups on day 16 or 41, as well as for the comparisons of histopathologic analyses between treatment groups. GraphPad Prism 11 (GraphPad Software, Inc.) was used for statistical analysis, and a *P* value < 0.05 was considered significant. The primary aim was the comparison of trabectedin versus the novel ecteinascidins, and additional comparisons are exploratory.

## Results

### PDX models showed stable histologic and molecular characteristics

All six established PDX models showed the key characteristics comparable with those found in their respective original patient samples (Supplementary Fig. S1), consistently exhibiting subtype-specific histopathologic and molecular features as previously described (23).

### Tumor volume assessment showed marked antitumor effect of novel DNA minor-groove binders *in vivo*

In all models, vehicle treatment was associated with a statistically significant growth on the final day of the experiment compared with day 1 (Supplementary Fig. S2A–S2G). In the sarcoma models with a more complex karyotype (DDLPS and LMS), ecubectedin and PM54 showed modest antitumor effects, with non–statistically significant differences compared with active control compounds (doxorubicin, trabectedin, and lurbinectedin; Table 1). The number of tumors included in the analysis is shown in Supplementary Table S1.

**Table 1.** Tumor volume assessment in STS models after treatment.

Xenograft model	Relative tumor volume [mean% (95% CI)]					
	Vehicle	Doxorubicin	Trabectedin	Lurbinectedin	Ecubectedin	PM54
STS111^LMS^	↑169*	↑170	128	↑132	= 101*	↑105
	(140–197)	(136–203)	(101–156)	(117–146)	(85–116)	(68–142)
STS22_2^LMS^	↑379	↑278	↑238	↑218	207	190
	(149–608)	(144–412)	(195–282)	(158–278)	(83–331)	(31–349)
STS204^DDLPS^	↑205*	↑148*	↑171	↑173	↑139	↑145
	(184–226)	(127–169)	(150–191)	(155–191)	(111–167)	(128–162)
STS112^DDLPS^	↑270*	↑184*	119	↑176*	113	↑134
	(216–324)	(137–231)	(95–144)	(149–203)	(89–137)	(110–158)
STS134^CRS^	↑387*	↑177	↑222	↑202	↓37*	↓76*
	(290–484)	(144–209)	(155–289)	(139–266)	(30–44)	(63–89)
STS336^SynSa^	↑259*	↑161	↑167	↑174	= 104*	= 100*
Day 16	(222–295)	(147–174)	(137–198)	(152–195)	(75–132)	(75–124)
STS336^SynSa^	↑439	↑269	↑414	↑376	= 101*	= 136*
Day 41	(247–631)	(207–331)	(224–605)	(309–442)	(64–138)	(52–220)

Table shows the mean relative tumor volume (%), with 95% CI. Arrows indicate whether the absolute tumor volume (not shown) was higher (↑), lower (↓), or not statistically different (=) compared with the starting volume, calculated using the Wilcoxon matched-pairs test. Statistical significance shown is related to the trabectedin-treated groups, calculated using the Mann–Whitney U test. *P < 0.05.

Abbreviation: CI, confidence interval.

**Figure 1.**
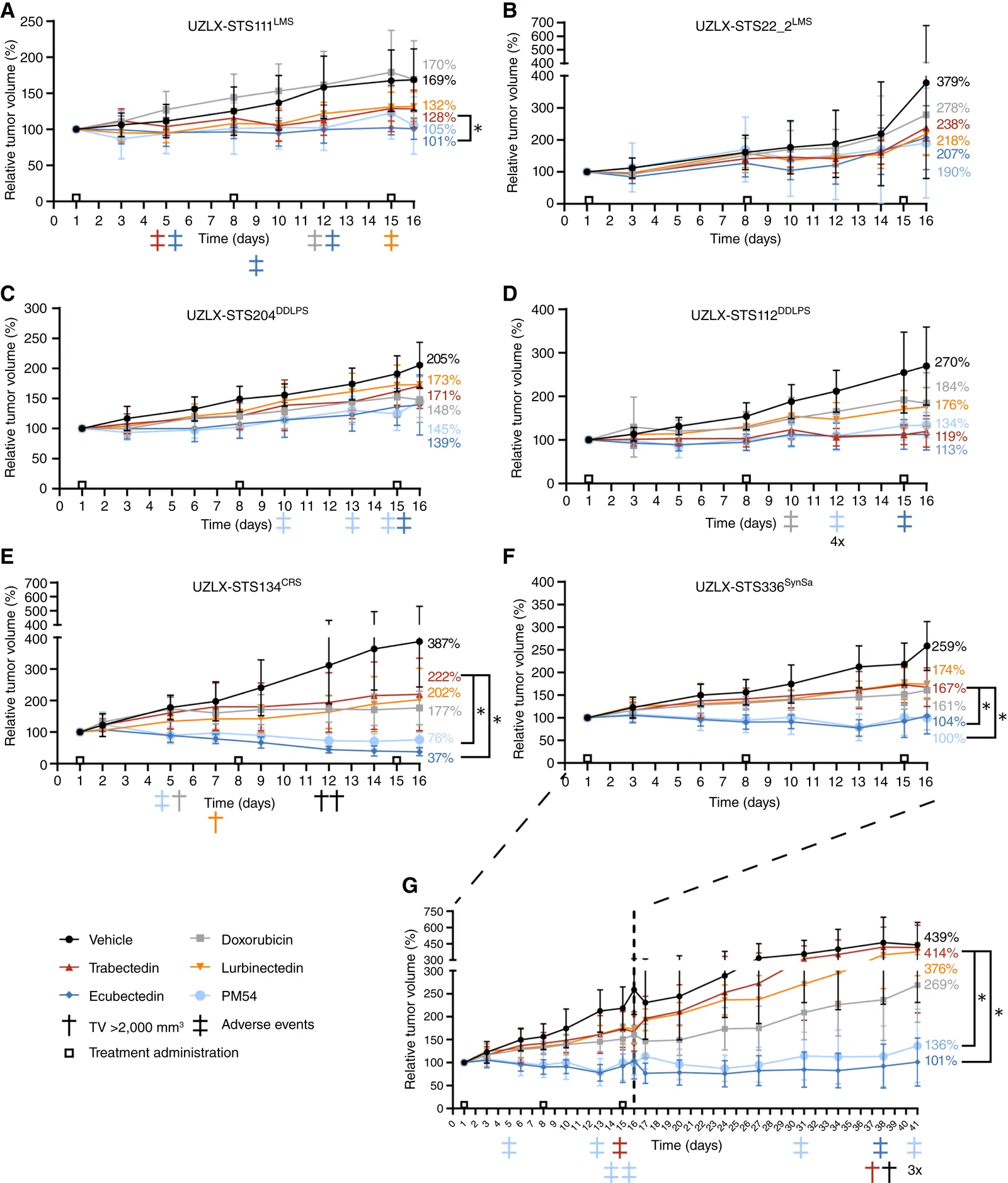
**A–G,** Evolution of tumor volume during treatment, presented as relative tumor volume (% change from normalized baseline) ± standard deviation. **F,** The first 16 days of the STS336^SynSa^ experiment. **G,** The complete experiment, including the follow-up period after the first 16 days. Tumors from mice sacrificed prior to the end of the experiments were not included in statistical evaluation. Statistical significance is indicated for ecubectedin or PM54 compared with trabectedin at the end of the experiment (day 16 or 41). *, *P* < 0.05. 3×/4×: 3 or 4 mice of the same group were sacrificed on the same day. TV, tumor volume.

In STS111^LMS^ (Fig. 1A), at the end of the experiment, ecubectedin-treated tumors were significantly smaller compared with those treated with trabectedin (*P* = 0.0411). PM54-treated tumors showed a lower average relative tumor volume compared with trabectedin (105% vs. 128%) yet showed no statistical significance (*P* > 0.05). In the other LMS model, STS22_2^LMS^, both ecubectedin and PM54 treatments were associated with tumor volume stabilization shown as non–statistically significant tumor growth (Supplementary Fig. S2B); however, it was not significantly better than the effect caused by other active controls (Fig. 1B).

Relative tumor volumes of ecubectedin- and PM54-treated tumors in STS204^DDLPS^ (139% and 145%, respectively) were not significantly smaller compared with trabectedin-treated tumors (171%, *P* > 0.05; Fig. 1C). Moreover, in STS112^DDLPS^, PM54-treated tumors were nonsignificantly larger than trabectedin-treated tumors (134% vs. 119%, *P* > 0.05; Fig. 1D).

In contrast to LMS and DDLPS models, tumor volume shrinkage was observed in STS134^CRS^ as a response to ecubectedin (63% regression from baseline; *P* < 0.0001) or PM54 (24% regression from baseline; *P* < 0.0001; Fig. 1E). At the same time, the active controls doxorubicin, trabectedin, and lurbinectedin showed statistically significant tumor growth (*P* = 0.0002, *P* = 0.0005 and *P* = 0.0024, respectively). Furthermore, in STS134^CRS^, ecubectedin and PM54 treatments resulted in significantly smaller tumors compared with trabectedin-treated tumors (*P* = 0.0001 for both drugs; Supplementary Fig. S3E). In STS336^SynSa^, the second translocation-related sarcoma model, both ecubectedin (104%) and PM54 (100%) showed tumor volume stabilization on day 16 (Fig. 1F) and statistically significantly smaller tumors compared with trabectedin (*P* = 0.0052 and *P* = 0.0031, respectively). Remarkably, this stabilization remained for up to 26 days after the final dose for both ecubectedin (101%) and PM54 (136%; Fig. 1G).

Overall, ecubectedin and PM54 showed modest, improved antitumor effects in the LMS and DDLPS models tested, compared with trabectedin (Supplementary Fig. S3A–S3G). However, both novel compounds led to significantly smaller tumors in both STS134^CRS^ and STS336^SynSa^ when compared with trabectedin.

### Histopathologic analysis corroborated the observed *in vivo* antitumor effect

**Figure 2.**
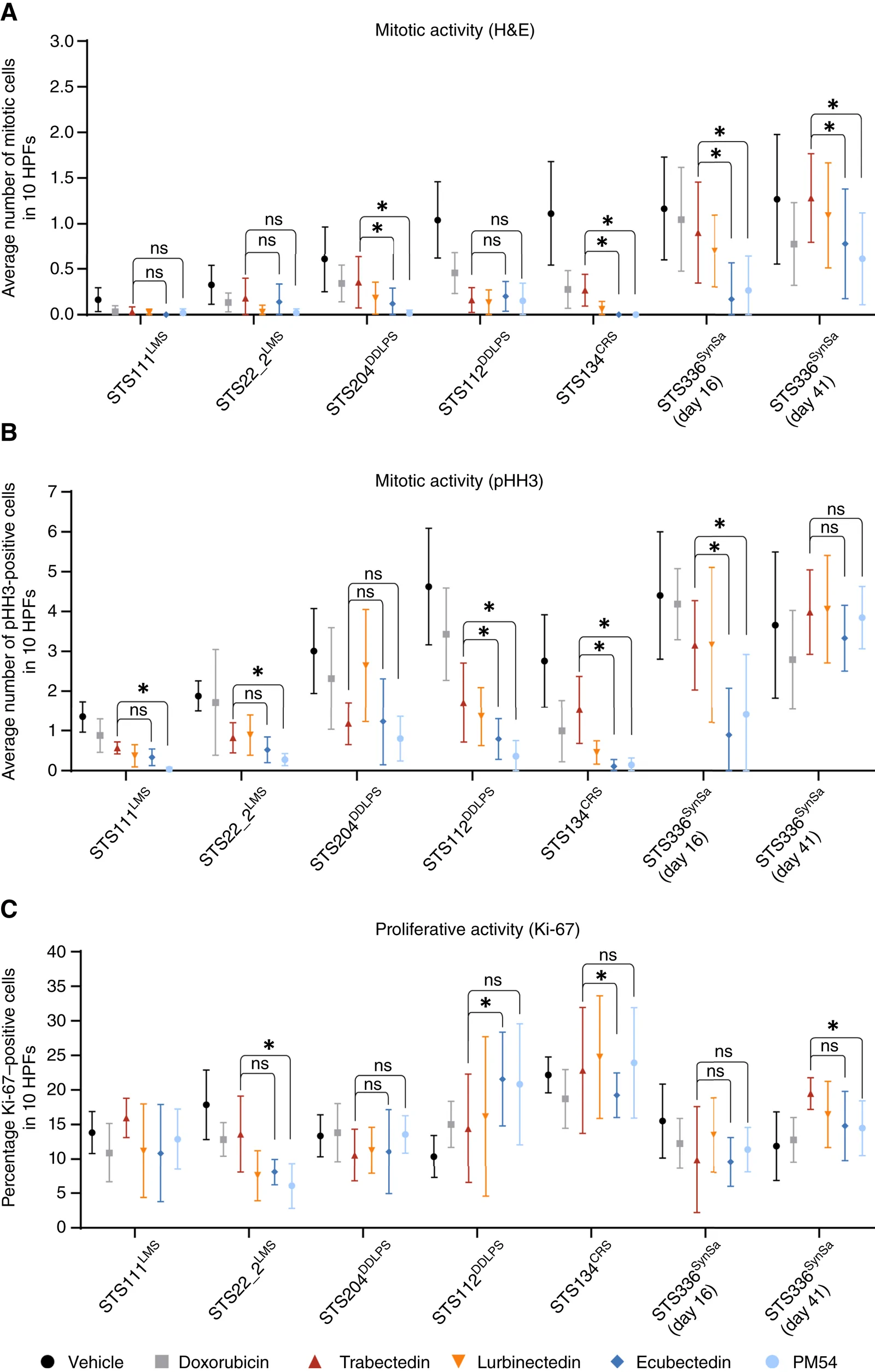
Average number of mitotic cells evaluated on (**A**) H&E and (**B**) mitotic activity shown by pHH3 IHC staining. **C,** Percentage of Ki-67–positive cells. The results show the mean ± standard deviation. Statistical significance was calculated using the Mann–Whitney U test, *P < 0.05. ns, not significant.

In all models, the mitotic activity was the highest in tumors treated with vehicle, on H&E- and on pHH3-stained slides (Fig. 2A–C). In STS111^LMS^, ecubectedin and PM54 showed a nonsignificant decrease in mitotic and proliferative activity when compared with trabectedin, with the exception of pHH3-stained slides in PM54-treated tumors (*P* = 0.0022). STS22_2^LMS^ showed a similar result, with decreased mitotic and proliferative activities in ecubectedin- and PM54-treated tumors compared with trabectedin. In STS204^DDLPS^, ecubectedin- and PM54-treated tumors showed a significant decrease in mitotic activity on H&E-stained slides (*P* = 0.0104 and *P* = 0.0001, respectively) compared with trabectedin, although this significance was not found when mitotic activity was assessed by pHH3 immunostaining. In contrast, the opposite was seen in the STS112^DDLPS^ model.

In the translocation-related sarcoma model STS134^CRS^, ecubectedin and PM54 caused a significant decrease in mitotic activity on both H&E- (*P* = 0.0004 and *P* = 0.0119, respectively) and pHH3-stained slides (*P* = 0.0007 and *P* = 0.039, respectively). STS336^SynSa^ showed a similar significant decrease in mitotic activity for ecubectedin- and PM54-treated tumors compared with trabectedin on day 16. Interestingly, on H&E, the mitotic activity of STS336^SynSa^ was still significantly lower on day 41 for both novel drugs when compared with trabectedin.

**Figure 3.**
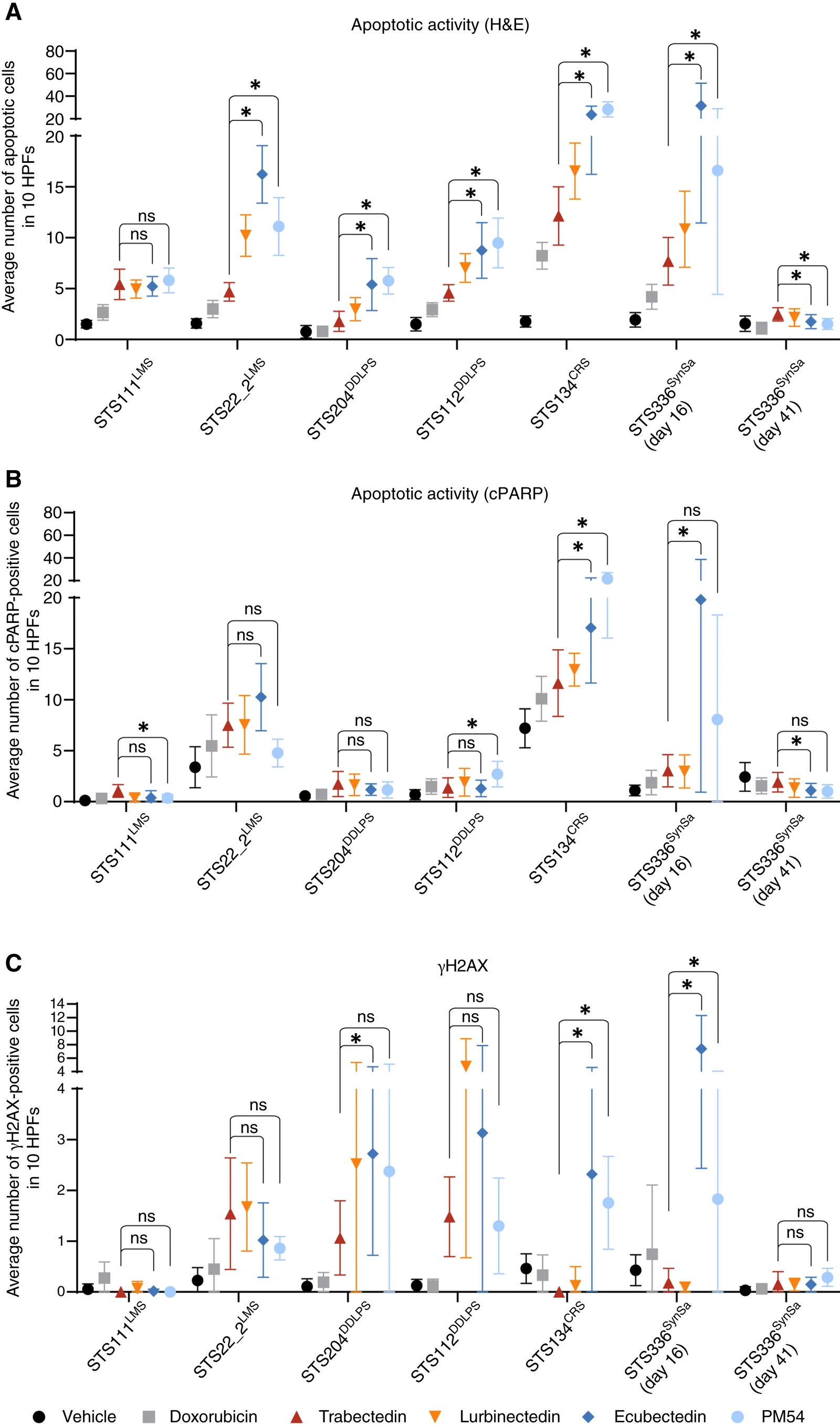
Average number of apoptotic cells evaluated on (**A**) H&E and (**B**) cPARP IHC staining. **C,** Double-strand break formation shown by γH2AX IHC staining. The results show the mean ± standard deviation. Statistical significance was calculated using the Mann–Whitney U test, *P < 0.05. ns, not significant.

To further validate the antitumor activity, the apoptotic activity was evaluated using H&E and cPARP (Fig. 3A and B). With the exception of STS111^LMS^, all LMS and DDLPS models tested showed a significant increase in apoptotic activity on H&E when treated with either ecubectedin or PM54, compared with trabectedin. These significant changes were, however, not reflected in the cPARP analysis for these subtypes. Both translocation-related sarcoma models showed a marked increase in antitumor activity when apoptotic activity was investigated. On H&E, ecubectedin and PM54 treatments resulted in a significant increase in apoptotic cells on day 16 in STS134^CRS^ (*P* = 0.001 and *P* = 0.0001, respectively) and STS336^SynSa^ (*P* = 0.0001 and *P* = 0.012, respectively). Analysis of the proapoptotic marker cPARP showed a similar significant increase compared with trabectedin for both ecubectedin and PM54 in STS134^CRS^ (*P* = 0.0058 and *P* = 0.0001, respectively). Meanwhile, only ecubectedin caused a significant increase in cPARP compared with trabectedin in STS336^SynSa^ on day 16 (*P* = 0.0445).

To investigate the extent of DNA damage, the DNA double-strand break marker γH2AX was analyzed (Fig. 3C). In the LMS and DDLPS models, only ecubectedin showed a significant increase in double-strand breaks in STS204^DDLPS^ (*P* = 0.0108). At the same time, both experimental compounds caused a significant increase in γH2AX-positive cells in STS134^CRS^ (*P* = 0.0001 for both) and STS336^SynSa^ (*P* = 0.0001 and *P* = 0.0047, respectively) on day 16 when compared with trabectedin.

Overall, the histopathologic analysis showed that ecubectedin and PM54 had a greater antitumor effect compared with trabectedin, a difference which was more pronounced in the translocation-related sarcoma models STS134^CRS^ and STS336^SynSa^ compared with the LMS and DDLPS models (Supplementary Table S2).

### The toxicity profile of novel ecteinascidins was comparable with trabectedin

Six mice were sacrificed because of reaching the humane endpoint of a tumor volume >2,000 mm^3^: three mice in the vehicle group, and one mouse each in the doxorubicin, trabectedin, and lurbinectedin groups (Supplementary Fig. S4A–S4G; Supplementary Table S3). In both doxorubicin (*n* = 49) and trabectedin (*n* = 53) groups, two mice each were sacrificed because of body weight loss. Trabectedin-treated mice showed a significantly lower body weight on day 16 of the STS336^SynSa^ experiment compared with ecubectedin- (*P* = 0.0216) and PM54-treated mice (*P* = 0.019). One of 51 mice in the lurbinectedin group suffered from edema, although this was considered unrelated to the treatment. Tail swelling was the most commonly noticed toxicity and occurred mainly in ecubectedin- (44/54, 81%), trabectedin- (31/53, 58%), and PM54-treated mice (32/55, 58%) and led to wound formation at the site of injection in 24 of 44 (55%), 9 of 31 (29%), and 15 of 32 (47%) of ecubectedin-, trabectedin-, and PM54-treated mice, respectively. Exacerbation of tail wound formation led to the sacrification of 4 of 54 mice (7%) in the ecubectedin group, two of which showed tail necrosis. Additionally, two mice had to be sacrificed because of body weight loss. Of the PM54-treated mice (*n* = 55), eight (15%) were sacrificed prematurely because of body weight loss, five of which showed exacerbated tail swelling and local wound formation. Additionally, two mice were sacrificed because of tail necrosis. Moreover, two mice were found dead, having shown a decrease in body weight prior to their finding. In the STS134^CRS^ experiment, PM54-treated mice showed significantly lower body weight compared with trabectedin (*P* = 0.0025). One mouse was sacrificed during follow-up of the STS336^SynSa^ experiment because of tail wounds which caused partial loss of the tail. In the STS336^synsa^ experiment, tails that showed wound formation during the treatment phase caused by any of the ecteinascidins did not recover, nor worsen, during the follow-up phase of that experiment. However, the tails became more rigid, and three PM54-treated mice were sacrificed, ending the experiment.

## Discussion

In the current study, we investigated the antitumor activity of two novel synthetic ecteinascidins, ecubectedin and PM54, in six PDX models of STS. Mice were transplanted bilaterally, based on an analysis previously performed by our group which proved that two tumors transplanted on the same mouse have a poor correlation and that this would have a negligible effect on the statistical evaluations of experiments such as those presented here (23).

Trabectedin is approved in the United States for the treatment of patients with LMS and LPS after failure of anthracyclines and ifosfamide (12). Trabectedin has previously shown activity in adipocytic sarcoma and LMS in a phase III trial (13). In the current project, LMS (STS111 and STS22_2) and DDLPS (STS112 and STS204) models treated with ecubectedin and PM54 showed tumor growth delay, similarly to trabectedin and lurbinectedin. The histopathologic analysis showed a modest decrease in mitotic activity and a strong increase in apoptotic activity on H&E, compared with trabectedin and lurbinectedin.

In contrast, the most striking response was observed in the translocation-related sarcoma model STS134^CRS^, an ultrarare sarcoma subtype which is very aggressive and difficult to treat (26). Both ecubectedin and PM54 caused significant tumor shrinkage after three doses (a decrease of 63% and 24% from baseline, respectively). Furthermore, the histopathologic analysis showed significant antitumor activity, evidenced by reduced mitosis and increased apoptosis in tumors treated with either novel compound, compared with trabectedin. When trabectedin was originally investigated in STS, a retrospective analysis found peculiar sensitivity in myxoid liposarcoma, a subtype of liposarcoma associated with *DDIT3::FUS* or *DDIT3::EWSR1* fusions, providing a first suggestion of trabectedin activity in translocation-related sarcoma (27).

In 2014, a randomized phase III trial investigated the use of trabectedin as first-line treatment, compared with doxorubicin, in translocation-related sarcoma, including 24 patients with SynSa (28). The rationale for the trial was the hypothesis that trabectedin may have preferential activity in these sarcomas, with evidence found in retrospective studies (15, 27). The trial did not demonstrate improved progression-free survival, although the investigators noted that the study was underpowered due to a high rate of censoring. A later retrospective study in heavily pretreated patients with SynSa supported the notion that trabectedin is active in this subpopulation (14). This knowledge, together with the impressive result in the STS134^CRS^ model, prompted us to adapt the setup of the STS336^SynSa^ experiment, increasing the cohort to include a follow-up period without treatment. On day 16, ecubectedin- and PM54-treated tumors showed tumor volume stabilization, and this effect remained for up to 26 days after the last dose was administered, when the experiment was terminated. Upon microscopic evaluation, some tumors treated with both novel ecteinascidins showed some alteration of the myxoid stroma on day 16 (Supplementary Fig. S5A–S5F). Review by an expert pathologist (R. Sciot) concluded that this alteration was not strong enough to classify as myxoid degeneration, a phenomenon frequently seen in gastrointestinal stromal tumors treated with imatinib. Nevertheless, this alteration could partially explain the lack of tumor shrinkage but rather tumor volume stabilization, which is considered a clinically relevant outcome in the current clinical landscape of STS (29).

Previous research has shown that the DNA repair profile plays an important role in the antitumor activity of trabectedin, specifically the activity of the transcription-coupled nucleotide excision repair system to create single-strand DNA breaks (8, 10). In addition, trabectedin-related double-strand break generation relies on deficiencies in the homologous recombination repair pathway (30). Furthermore, a retrospective study by Schöffski and colleagues (31) found a signature of expression of three genes (*BRCA1*^*low*^, *ERCC1*^*high*^, and *XPG*^*high*^) to have predictive value of trabectedin sensitivity in sarcoma. However, to date, this signature has not been validated in clinical practice for the selection of patients for treatment with trabectedin. Additionally, the relationship between dysfunctional homologous recombination repair and the novel ecteinascidins is as of yet unproven. Given this information, as well as the heterogenous responses seen in the different sarcoma models tested, we deem further exploration of the expression of DNA repair mechanisms of the models necessary, a project which is currently ongoing. We will perform a gene set enrichment analysis on RNA extracted from untreated tumor material of the presently described models, focusing on DNA damage repair, and correlate these findings with our *in vivo* results in an attempt to further explain the striking differences in treatment response. Additionally, these results may inform further development of the novel ecteinascidins and reveal important differences between the now four generations of these compounds. Finally, we will examine transcriptional changes elicited by ecteinascidin treatment, comparing with vehicle-treated samples.

Trabectedin has shown a manageable toxicity profile in humans in clinical routine (32). Overall, ecubectedin- and PM54-treated mice showed similar types of toxicity compared with mice treated with either trabectedin or lurbinectedin. The most notable toxicity was swelling and wound formation at the site of injection in the tail of the mice, most pronounced in trabectedin-, ecubectedin-, and PM54-treated animals. During the experiments, we noticed that wound formation only occurred when the drugs were administered inaccurately, outside the tail vein. Even without the noted inaccurate drug administration, we did observe tail swelling, albeit to a much lesser degree. Of the surviving cohort, PM54-treated mice only showed a significantly decreased body weight in STS134^CRS^. However, in mice with tail-related toxicities (e.g., tail swelling), we noticed that substantially more PM54-treated mice were lost (9/32, 28%) compared with ecubectedin- (4/44, 9%) and trabectedin-treated mice (1/31, 3%). We hypothesize that some extravasation of the drug can occur as a result of vasodilation caused by the warming of the tail prior to injection, which is a standard procedure for intravenous injections in murine experiments. Tail swelling and wound formation are known to occur in murine experiments with this class of drug, in which the most viable method of treatment administration is via the tail vein (33). Interestingly, mice included in the follow-up phase of the STS336^SynSa^ experiment did not show continuous deterioration of tail wounds after the last treatment. However, we observed that the flexibility of the tails did decrease, likely due to wound healing or fibrosis after treatment cessation. The weight loss in the ecubectedin and PM54 groups can at least partially be attributed to the damage caused to the tails, causing the mice to be unable to reach food, placed up high in the racks covering the cages, easily and free of pain. In patients, trabectedin is administered via a central venous line, and extravasation of trabectedin at the site of injection is known to give an inflammatory reaction (34). Future experiments should take into account this adverse event, providing alternative feeding methods for mice throughout experiments, such as gel cups placed on cage floors for ease of access. An alternative option would be to place central venous catheters in mice (35). However, this technique does require specialized training and equipment and is very time-consuming in larger cohorts such as those used in our current experiment. In smaller cohorts, such route of administration could be considered, especially in long-term drug exposure studies in mice.

In the current study, we have shown that ecubectedin and PM54 have antitumor activity in selected subtypes of STS, when given as monotherapy. Although trabectedin is generally given as monotherapy in clinical practice, there are some studies evaluating combination therapies. A recent study by Pautier and colleagues (36) has investigated doxorubicin plus trabectedin followed by trabectedin maintenance in patients with metastatic LMS in the first line. Patients in the combination group showed longer progression-free survival (12 months versus 6 months in the doxorubicin monotherapy group). A similar currently ongoing phase IIb/III study is investigating the combination of lurbinectedin and doxorubicin with lurbinectedin maintenance in the first line (NCT06088290; ref. 37). Another combination therapy under investigation is trabectedin with PARP inhibitors (e.g., olaparib; ref. 38). A recently completed phase II study found limited improvement in patients treated with trabectedin and olaparib versus trabectedin alone (median progression-free survival 3.9 months versus 2.9 months, respectively; ref. 39). However, the investigators noted that patients with tumors with high expression of PARP1 showed a significantly higher objective response rate (27% versus 8% in PARP1-high versus -low; ref. 39).

### Conclusion

The present study explored the antitumor activity of ecubectedin and PM54, two novel synthetic ecteinascidins, in six well-characterized PDX models of STS. Ecubectedin and PM54 showed modest anticancer effect in LMS and DDLPS. In contrast, the treatment with the novel ecteinascidins of the two translocation-related sarcoma models led to a strong antitumor response, building upon previous data that this sarcoma subgroup has an inherent, not yet fully understood disposition to treatment with this class of drugs. In conclusion, ecubectedin and PM54 show favorable anticancer activity in STS, warranting additional preclinical and clinical investigation.

## Supplementary Material

Supplemental Figure 1 Supplemental Figure 1:Patient-derived xenograft model characterization: alpha-SMA: alpha smooth muscle actin; CD99: cluster of differentiation 99; CRS: CIC-rearranged sarcoma; DDLPS: dedifferentiated liposarcoma; EMA: epithelial membrane antigen; FISH: fluorescent in situ hybridization; HLA-A: human leukocyte antigen-A; H&E hematoxylin & eosin; LMS: leiomyosarcoma; MDM2: mouse double minute homolog 2; SynSa: synovial sarcoma; TLE-1: transducing-like enhancer of split 1; UZLX: University Hospitals Leuven xenograft.

Supplemental Figure 2 Supplemental Figure 2: Evolution of absolute tumor volume of individual tumors from day 1 to day 16 (A-F) or from day 1 to day 17 and to day 41 (G). Statistical significance indicate either tumor growth or shrinkage, whereas non-significance indicates tumor volume stabilization. * p<0.05, CRS: CIC-rearranged sarcoma; DDLPS: dedifferentiated liposarcoma; LMS: leiomyosarcoma; STS: soft tissue sarcoma; TV: tumor volume; ns: not significant; SynSa: synovial sarcoma.

Supplemental Figure 3 Supplemental Figure 3: Relative change of individual tumor volumes from baseline (here indicated as 0), on day 16 (A-F) or day 41 (G), sorted per treatment for each experiment.

Supplemental Figure 4 Supplemental Figure 4: Evolution of body weight during treatment, presented as relative body weight (% change from normalized baseline) ± standard deviation. Figure 2F depicts the first phase of the experiment of STS336SynSa, while Figure 2G depicts the complete experiment. Mice sacrificed prior to end of experiment are removed from data after date of scarification. 3x/4x: 3 or 4 mice of the same group were sacrificed on the same day; * p<0.05; CRS: CIC-rearranged sarcoma; DDLPS: dedifferentiated liposarcoma; LMS: leiomyosarcoma; ns: not significant; STS: soft tissue sarcoma; TV: tumor volume; SynSa: synovial sarcoma.

Supplemental Figure 5 Supplemental Figure 5: Overview of representative tumor sections of STS336SynSa on day 16, treated with A) vehicle, B) doxorubicin, C) trabectedin, D) lurbinectedin, E) ecubectedin, and F) PM54. Tumors treated with ecubectedin and PM54 showed alteration in the myxoid stroma after treatment, potentially explaining the absence of tumor volume shrinkage despite the increase in mitosis and decrease in apoptosis. SynSa: synovial sarcoma.

Supplemental Table 1 List of treatment groups, models, number of tumors included at the start of the experiment and the number of tumors included in the analysis. Tumors from mice that were sacrificed prior to the end of the experiment (e.g., due to unethical tumor volume >2000mm 3 or body weight loss), were not included in the analysis. Tumors excluded from analysis of phase 2 of the STS336SynSa experiment concerns tumors from mice designated to the group intended for follow-up at any point in the experiment (i.e., any moment between days 0 and 41). CRS: CIC-rearranged sarcoma; DDLPS: dedifferentiated liposarcoma; LMS: leiomyosarcoma; SynSa: synovial sarcoma

Supplemental Table 2 Evaluation of mitotic and apoptotic activity Arrows indicate whether mitotic or apoptotic activity was significantly higher (↑), lower (↓) or not statistically different (=) compared to trabectedin. Statistically significant changes were calculated using the Mann-Whitney U test, compared to trabectedin. CRS: CIC-rearranged sarcoma; DDLPS: dedifferentiated liposarcoma; H&E: hematoxylin and eosin; LMS; leiomyosarcoma; SynSa; synovial sarcoma

Supplemental Table 3 Loss of mice before the end of the experiment and reason of sacrification. CRS: CIC-rearranged sarcoma; DDLPS: dedifferentiated liposarcoma; LMS: leiomyosarcoma; SynSa: synovial sarcoma.

## Data Availability

The data generated in this study are available upon request to the corresponding author.

## References

[1] Damerell V , Pepper MS, Prince S. Molecular mechanisms underpinning sarcomas and implications for current and future therapy. Signal Transduct Target Ther 2021;6:246. 10.1038/s41392-021-00647-8. 34188019 PMC8241855

[2] WHO Classification of Tumours Editorial Board . Soft tissue and bone tumours. 5th ed. Lyon (France): International Agency for Research on Cancer (IARC); 2020.

[3] Cancer Genome Atlas Research Network . Comprehensive and integrated genomic characterization of adult soft tissue sarcomas. Cell 2017;171:950–65.e28. 10.1016/j.cell.2017.10.014. 29100075 PMC5693358

[4] Nakano K , Takahashi S. Translocation-related sarcomas. Int J Mol Sci 2018;19:3784. 10.3390/ijms19123784. 30487384 PMC6320865

[5] Stiller CA , Trama A, Serraino D, Rossi S, Navarro C, Chirlaque MD, . Descriptive epidemiology of sarcomas in Europe: report from the RARECARE project. Eur J Cancer 2013;49:684–95. 10.1016/j.ejca.2012.09.011. 23079473

[6] Judson I , Verweij J, Gelderblom H, Hartmann JT, Schöffski P, Blay JY, . Doxorubicin alone versus intensified doxorubicin plus ifosfamide for first-line treatment of advanced or metastatic soft-tissue sarcoma: a randomised controlled phase 3 trial. Lancet Oncol 2014;15:415–23. 10.1016/S1470-2045(14)70063-4. 24618336

[7] Pommier Y , Kohlhagen G, Bailly C, Waring M, Mazumder A, Kohn KW. DNA sequence- and structure-selective alkylation of guanine N2 in the DNA minor groove by ecteinascidin 743, a potent antitumor compound from the Caribbean tunicate Ecteinascidia turbinata. Biochemistry 1996;35:13303–9. 10.1021/bi960306b. 8873596

[8] Takebayashi Y , Pourquier P, Zimonjic DB, Nakayama K, Emmert S, Ueda T, . Antiproliferative activity of ecteinascidin 743 is dependent upon transcription-coupled nucleotide-excision repair. Nat Med 2001;7:961–6. 10.1038/91008. 11479630

[9] Fousteri M , Mullenders LH. Transcription-coupled nucleotide excision repair in mammalian cells: molecular mechanisms and biological effects. Cell Res 2008;18:73–84. 10.1038/cr.2008.6. 18166977

[10] Son K , Takhaveev V, Mor V, Yu H, Dillier E, Zilio N, . Trabectedin derails transcription-coupled nucleotide excision repair to induce DNA breaks in highly transcribed genes. Nat Commun 2024;15:1388. 10.1038/s41467-024-45664-7. 38360910 PMC10869700

[11] Caldecott KW . Causes and consequences of DNA single-strand breaks. Trends Biochem Sci 2024;49:68–78. 10.1016/j.tibs.2023.11.001. 38040599

[12] Demetri GD , von Mehren M, Jones RL, Hensley ML, Schuetze SM, Staddon A, . Efficacy and safety of trabectedin or dacarbazine for metastatic liposarcoma or leiomyosarcoma after failure of conventional chemotherapy: results of a phase III randomized multicenter clinical trial. J Clin Oncol 2016;34:786–93. 10.1200/JCO.2015.62.4734. 26371143 PMC5070559

[13] Demetri GD , Chawla SP, von Mehren M, Ritch P, Baker LH, Blay JY, . Efficacy and safety of trabectedin in patients with advanced or metastatic liposarcoma or leiomyosarcoma after failure of prior anthracyclines and ifosfamide: results of a randomized phase II study of two different schedules. J Clin Oncol 2009;27:4188–96. 10.1200/JCO.2008.21.0088. 19652065

[14] Sanfilippo R , Dileo P, Blay JY, Constantinidou A, Le Cesne A, Benson C, . Trabectedin in advanced synovial sarcomas: a multicenter retrospective study from four European institutions and the Italian Rare Cancer Network. Anticancer Drugs 2015;26:678–81. 10.1097/CAD.0000000000000228. 25763543 PMC4730787

[15] Le Cesne A , Cresta S, Maki RG, Blay JY, Verweij J, Poveda A, . A retrospective analysis of antitumour activity with trabectedin in translocation-related sarcomas. Eur J Cancer 2012;48:3036–44. 10.1016/j.ejca.2012.05.012. 22749255

[16] Leal JFM , Martínez-Díez M, García-Hernández V, Moneo V, Domingo A, Bueren-Calabuig JA, . PM01183, a new DNA minor groove covalent binder with potent in vitro and in vivo anti-tumour activity. Br J Pharmacol 2010;161:1099–110. 10.1111/j.1476-5381.2010.00945.x. 20977459 PMC2998690

[17] Trigo J , Subbiah V, Besse B, Moreno V, López R, Sala MA, . Lurbinectedin as second-line treatment for patients with small-cell lung cancer: a single-arm, open-label, phase 2 basket trial. Lancet Oncol 2020;21:645–54. 10.1016/S1470-2045(20)30068-1. 32224306

[18] Gaillard S , Oaknin A, Ray-Coquard I, Vergote I, Scambia G, Colombo N, . Lurbinectedin versus pegylated liposomal doxorubicin or topotecan in patients with platinum-resistant ovarian cancer: a multicenter, randomized, controlled, open-label phase 3 study (CORAIL). Gynecol Oncol 2021;163:237–45. 10.1016/j.ygyno.2021.08.032. 34521554

[19] Singh S , Jaigirdar AA, Mulkey F, Cheng J, Hamed SS, Li Y, . FDA approval summary: lurbinectedin for the treatment of metastatic small cell lung cancer. Clin Cancer Res 2021;27:2378–82. 10.1158/1078-0432.CCR-20-3901. 33288660 PMC9532454

[20] Cote GM , Choy E, Chen T, Marino-Enriquez A, Morgan J, Merriam P, . A phase II multi-strata study of lurbinectedin as a single agent or in combination with conventional chemotherapy in metastatic and/or unresectable sarcomas. Eur J Cancer 2020;126:21–32. 10.1016/j.ejca.2019.10.021. 31896519

[21] Cigrang M , Obid J, Nogaret M, Seno L, Ye T, Davidson G, . Pan-inhibition of super-enhancer-driven oncogenic transcription by next-generation synthetic ecteinascidins yields potent anti-cancer activity. Nat Commun 2025;16:512. 10.1038/s41467-024-55667-z. 39779693 PMC11711318

[22] Salaroglio IC , Aviles P, Kopecka J, Merlini A, Napoli F, Righi L, . Ecteinascidin synthetic analogues: a new class of selective inhibitors of transcription, exerting immunogenic cell death in refractory malignant pleural mesothelioma. J Exp Clin Cancer Res 2024;43:327. 10.1186/s13046-024-03253-y. 39709435 PMC11662834

[23] Cornillie J , Wozniak A, Li H, Wang Y, Boeckx B, Gebreyohannes YK, . Establishment and characterization of histologically and molecularly stable soft-tissue sarcoma xenograft models for biological studies and preclinical drug testing. Mol Cancer Ther 2019;18:1168–78. 10.1158/1535-7163.MCT-18-1045. 30962320

[24] Gebreyohannes YK , Burton EA, Wozniak A, Matusow B, Habets G, Wellens J, . PLX9486 shows anti-tumor efficacy in patient-derived, tyrosine kinase inhibitor-resistant KIT-mutant xenograft models of gastrointestinal stromal tumors. Clin Exp Med 2019;19:201–10. 10.1007/s10238-018-0541-2. 30523507

[25] Going JJ . Counting cells made easier. Histopathology 2006;49:309–11. 10.1111/j.1365-2559.2006.02458.x. 16918979

[26] Antonescu CR , Owosho AA, Zhang L, Chen S, Deniz K, Huryn JM, . Sarcomas with CIC-rearrangements are a distinct pathologic entity with aggressive outcome: a clinicopathologic and molecular study of 115 cases. Am J Surg Pathol 2017;41:941–9. 10.1097/PAS.0000000000000846. 28346326 PMC5468475

[27] Grosso F , Jones RL, Demetri GD, Judson IR, Blay JY, Le Cesne A, . Efficacy of trabectedin (ecteinascidin-743) in advanced pretreated myxoid liposarcomas: a retrospective study. Lancet Oncol 2007;8:595–602. 10.1016/S1470-2045(07)70175-4. 17586092

[28] Blay JY , Leahy MG, Nguyen BB, Patel SR, Hohenberger P, Santoro A, . Randomised phase III trial of trabectedin versus doxorubicin-based chemotherapy as first-line therapy in translocation-related sarcomas. Eur J Cancer 2014;50:1137–47. 10.1016/j.ejca.2014.01.012. 24512981

[29] Grünwald V , Litière S, Young R, Messiou C, Lia M, Wardelmann E, . Absence of progression, not extent of tumour shrinkage, defines prognosis in soft-tissue sarcoma - an analysis of the EORTC 62012 study of the EORTC STBSG. Eur J Cancer 2016;64:44–51. 10.1016/j.ejca.2016.05.023. 27323349

[30] Guirouilh-Barbat J , Redon C, Pommier Y. Transcription-coupled DNA double-strand breaks are mediated via the nucleotide excision repair and the Mre11-Rad50-Nbs1 complex. Mol Biol Cell 2008;19:3969–81. 10.1091/mbc.e08-02-0215. 18632984 PMC2526702

[31] Schöffski P , Taron M, Jimeno J, Grosso F, Sanfilipio R, Casali PG, . Predictive impact of DNA repair functionality on clinical outcome of advanced sarcoma patients treated with trabectedin: a retrospective multicentric study. Eur J Cancer 2011;47:1006–12. 10.1016/j.ejca.2011.01.016. 21376569

[32] Grignani G , Le Cesne A, Martín-Broto J. Trabectedin as second-line treatment in advanced soft tissue sarcoma: quality of life and safety outcomes. Future Oncol 2022;18:13–22. 10.2217/fon-2022-0518. 36200954

[33] Bello E , Brich S, Craparotta I, Mannarino L, Ballabio S, Gatta R, . Establishment and characterisation of a new patient-derived model of myxoid liposarcoma with acquired resistance to trabectedin. Br J Cancer 2019;121:464–73. 10.1038/s41416-019-0550-2. 31409911 PMC6738121

[34] Schöffski P , Cerbone L, Wolter P, De Wever I, Samson I, Dumez H, . Administration of 24-h intravenous infusions of trabectedin in ambulatory patients with mesenchymal tumors via disposable elastomeric pumps: an effective and patient-friendly palliative treatment option. Onkologie 2012;35:14–7. 10.1159/000335879. 22310339

[35] Ibrahim N , Klopf J, Bleichert S, Bailey MA, Busch A, Stiglbauer-Tscholakoff A, . Drug treatment by central venous catheter in a mouse model of angiotensin II induced abdominal aortic aneurysm and monitoring by 3D ultrasound. J Vis Exp 2022;186:1–14. 10.3791/64124. 35993760

[36] Pautier P , Italiano A, Piperno-Neumann S, Chevreau C, Penel N, Firmin N, . Doxorubicin-trabectedin with trabectedin maintenance in leiomyosarcoma. N Engl J Med 2024;391:789–99. 10.1056/NEJMoa2403394. 39231341

[37] Cote GM , Chawla SP, Demetri G, Kasper B, Jones RL, Broto JM, . SaLudo: a randomized phase IIb/III study of lurbinectedin plus doxorubicin as first-line treatment in leiomyosarcoma. Future Oncol 2025;21:943–51. 10.1080/14796694.2025.2463798. 39932221 PMC11938983

[38] Pignochino Y , Capozzi F, D’Ambrosio L, Dell’Aglio C, Basiricò M, Canta M, . PARP1 expression drives the synergistic antitumor activity of trabectedin and PARP1 inhibitors in sarcoma preclinical models. Mol Cancer 2017;16:86. 10.1186/s12943-017-0652-5. 28454547 PMC5410089

[39] D’Ambrosio L , Merlini A, Brunello A, Ferraresi V, Paioli A, Vincenzi B, . Trabectedin-olaparib combination or trabectedin in advanced soft tissue sarcomas after failure of anthracycline-based treatment (TOMAS2): a randomized phase II study from the Italian Sarcoma Group. Ann Oncol 2026;37:521–31. 10.1016/j.annonc.2025.11.019. 41354211

[40] Gorgels D , Wang C-C, Wyns K, De Cock L, De Sutter L, Verbeeck K, . The novel transcriptional inhibitor ecubectedin demonstrates strong antitumor activity in patient-derived xenograft models of soft tissue sarcoma [abstract]. In: Proceedings of the American Association for Cancer Research Annual Meeting 2025; Part 1 (Regular Abstracts); 2025 Apr 25–30; Chicago, IL. Philadelphia (PA): AACR; Cancer Res 2025;85(8_Suppl_1):Abstract nr 6857.

[41] Gorgels D , Wang C-C, Wyns K, De Cock L, De Sutter L, Verbeeck K, . The next generation transcriptional inhibitor PM54 demonstrates strong antitumor activity in patient-derived xenograft models of soft tissue sarcoma [abstract]. In: Proceedings of the American Association for Cancer Research Annual Meeting 2025; Part 1 (Regular Abstracts); 2025 Apr 25–30; Chicago, IL. Philadelphia (PA): AACR; Cancer Res 2025;85(8_Suppl_1):Abstract nr 6858.

